# Connection between Genetic and Clinical Data in Bipolar Disorder

**DOI:** 10.1371/journal.pone.0044623

**Published:** 2012-09-20

**Authors:** Erling Mellerup, Ole Andreassen, Bente Bennike, Henrik Dam, Srdjan Durovic, Thomas Hansen, Ingrid Melle, Gert Lykke Møller, Ole Mors, Pernille Koefoed

**Affiliations:** 1 Laboratory of Neuropsychiatry, Department of Neuroscience and Pharmacology, University of Copenhagen, Blegdamsvej, Copenhagen, Denmark; 2 Psychiatric Centre Copenhagen, Department O, Copenhagen University Hospital, Rigshospitalet, Blegdamsvej, Copenhagen, Denmark; 3 Department of Psychiatry, Oslo University Hospital and Institute of Psychiatry, University of Oslo, Kirkeveien, Oslo, Norway; 4 Department of Medical Genetics, Oslo University Hospital and Institute of Psychiatry, University of Oslo, Kirkeveien, Oslo, Norway; 5 Department of Biological Psychiatry, Mental Health Centre Sct. Hans, Copenhagen University Hospital, Boserupvej 2, Roskilde, Denmark; 6 Centre for Psychiatric Research, Aarhus University Hospital, Skovagervej, Risskov, Denmark; 7 Genokey ApS, ScionDTU, Technical University Denmark, Agern Allé, Hoersholm, Denmark; University of Illinois at Chicago, United States of America

## Abstract

Complex diseases may be associated with combinations of changes in DNA, where the single change has little impact alone. In a previous study of patients with bipolar disorder and controls combinations of SNP genotypes were analyzed, and four large clusters of combinations were found to be significantly associated with bipolar disorder. It has now been found that these clusters may be connected to clinical data.

## Introduction

Modern analytical methods, particularly in the field of molecular genetics, produce large amounts of data that pose a challenge to statistical and data-mining methods for extracting useful information [1], [2]. Thus, in polygenic diseases finding disease related genetic changes, among the vast number of changes (e.g., millions of SNPs), is a daunting task. In a recent study [3], 803 SNPs in samples from 607 bipolar patients and 1355 control subjects were analyzed. All the SNPs were from genes selected based on theoretical and experimental studies that suggested that signal transduction and particular ion channels were involved in bipolar disorder [4]–[6]. The number of combinations of 3 SNP genotypes was counted in the material [3]. The theoretical number of combinations of 3 SNP genotypes taken from 803 SNPs is 2,321,319,627 (803!/3!(803 – 3)!×3^3)^, and as many as 1,985,613,130 combinations were found in the participants. 1,719,002,329 combinations were common between controls and patients, 208,699,590 combinations were found in controls only, and 57,911,211 combinations were found in patients only, of these 45,285,770 occurred only once, and not more than 1181 combinations were shared by 9 or more patients. None of the 803 single SNPs or the nearly two billion of SNP genotype combinations showed a statistically significant association with bipolar disorder. However, among the 1181 combinations shared by 9 or more patients and no controls, four clusters of combinations, were identified that were significantly associated with bipolar disorder. Within a cluster, each patient had a personal pattern of SNP genotypes that was somewhat similar to the patterns of other patients in the same cluster, but quite different from the patterns of patients in the other three clusters, hereby suggesting an extreme degree of genetic heterogeneity [3], [7].

## Results

The four clusters, are shown in Table 1, 2, 3, 4. Of the 607 patients, 156 were members of the 4 clusters. The clusters contained 41, 48, 41, and 37 patients; 11 patients were members of two clusters, and no patient was a member of three clusters. The clusters contained 60, 60, 65, and 53 SNP genotypes; 29 SNP genotypes were located in two clusters, and one SNP genotype (rs1380452 positioned in the *ANK3* gene) was located in three clusters.

**Table 1:** Cluster 1. defined by SNP1 = AVPR1B_rs33976516 = 1.

SNP2	SNP3	GT^a^	Patients^b^
ANK3_rs2288358	SCN2B_rs8192614	2 0	94 126 132 166 333 393 409 413 528
ANK3_rs2288358	CAMKK2_rs11065502	2 0	94 126 132 166 333 393 409 413 528
ANK3_rs2288358	PPP2R2C_rs17721365	2 0	94 126 132 166 333 393 409 413 528
ANK3_rs2288358	ANK3_rs10994322	2 0	94 126 132 166 333 393 409 413 528
ANK3_rs2288358	KCNC2_rs1880840	2 0	94 126 132 166 333 393 409 413 528
ANK3_rs4948255	KCNQ2_rs3787119	2 0	94 126 151 166 333 393 409 413 528
ANK3_rs4948255	KCNN3_rs12029542	2 0	94 126 151 166 333 393 409 413 528
ANK3_rs4948255	ATP1A3_rs4803520	2 0	94 126 132 151 166 333 393 409 528
ANK3_rs4948255	ATP1A3_rs2217342	2 0	94 126 132 151 166 333 393 409 528
ANK3_rs4948255	CACNG2_rs4821512	2 1	94 132 151 166 333 393 409 413 528
CNTNAP2_rs6945513	NFASC_rs17415523	0 0	105 149 197 200 210 231 278 333 390
CNTNAP2_rs6945513	ANK3_rs17805456	0 0	6 105 149 197 200 210 231 333 390
CNTNAP2_rs6945513	ANK3_rs7895653	0 0	6 105 149 200 210 231 278 333 390
CNTNAP2_rs6945513	ANK3_rs10994322	0 0	6 105 149 200 210 231 278 333 390
CNTNAP2_rs6945513	KCNN3_rs951241	0 0	6 105 149 200 210 231 278 333 390
KCNN3_rs6699080	SCN2B_rs8192614	2 0	6 94 100 126 151 278 285 366 409 593
KCNN3_rs6699080	TNR_rs2236885	2 0	6 94 100 126 151 278 285 366 409 593
KCNN3_rs6699080	ANK3_rs4359155	2 0	6 94 100 126 151 278 285 409 593
KCNN3_rs6699080	MCTP2_rs3784644	2 0	6 94 100 151 278 285 366 409 593
P2RX7_rs6489794	OLIG2_rs762178	1 1	42 151 166 210 248 330 393 421 511
P2RX7_rs6489794	CACNG2_rs2284016	1 0	57 151 248 330 356 366 393 421 511
P2RX7_rs6489794	ANK3_rs2393602	1 0	57 210 231 248 304 330 366 421 511
KCNN3_rs1218575	CREB1_rs2551921	1 1	57 126 151 200 210 231 393 436 511 593
KCNN3_rs1218575	CNTNAP2_rs2620460	1 1	126 151 200 231 248 393 409 436 545
KCNN3_rs1218575	ANK3_rs1010556	1 2	6 57 94 200 393 409 511 545 593
ANK3_rs10821695	TRPM2_rs1556314	0 1	42 94 114 231 285 333 393 421 575
ANK3_rs10821695	TRPM2_rs734336	0 1	42 94 114 166 231 285 333 393 575
NFASC_rs16854930	IMPA2_rs628419	1 1	51 94 132 248 390 393 394 511 575 593
NFASC_rs16854930	SCN1B_rs8100085	1 1	166 285 356 393 409 413 436 511 575
NCAM1_rs12794326	TNR_rs2239821	2 1	88 149 200 210 304 330 390 394 528 596
NCAM1_rs12794326	CNTN1_rs7315781	2 0	149 200 210 330 390 393 528 575 596
CNTN2_rs16855045	P2RX7_rs1718161	1 1	51 149 166 210 231 248 330 393 413 421 593
CNTN2_rs16855045	CACNG2_rs2283970	1 0	51 57 149 210 248 330 333 356 413 421
ANK3_rs1010556	ANK3_rs10994195	0 1	42 51 88 114 149 153 166 278 366 413 421
ANK3_rs1010556	CNTNAP2_rs1024676	2 0	6 57 94 132 304 409 511 545 593
KCNN3_rs6426998	TNR_rs1385541	2 0	6 94 126 278 285 393 409 421 593
KCNQ2_rs6122454	NRCAM_rs759548	0 1	57 149 200 285 304 356 421 511 545
SCN4B_rs868344	ANK3_rs7893313	2 1	35 42 88 94 100 149 333 356 409
ANK3_rs1380452	ANK3_rs10994171	2 0	6 57 100 105 132 304 409 436 511 593
SCN4B_rs678262	ANK3_rs2018783	0 2	42 94 100 114 248 304 393 409 436
CACNG2_rs2283970	CNTN2_rs3767298	0 1	51 57 149 210 248 333 356 413 421
P2RX7_rs1718161	CNTN2_rs3767298	1 1	51 114 149 166 210 231 248 393 413 421 593
ATP1A3_rs4803520	KCNN3_rs6426998	0 2	6 94 126 151 285 366 393 409 421
ANK3_rs17805456	CNTNAP2_rs10808044	0 2	6 105 149 153 197 210 231 366 390 413
NCAM1_rs584427	AQP4_rs3763043	2 0	35 151 200 366 390 409 413 421 436 545
NFASC_rs2802853	KCNQ3_rs869710	1 0	57 88 94 105 248 285 356 393 575 593

Cluster 1 contains 41 patients, 46 combinations of 3 SNP genotypes, and 58 SNP genotypes.

a) GT = genotype for SNP2 and SNP3 (0: Normal homozygote.1: Heterozygote. 2: Variant homozygote); b) Dummy ID.

**Table 2:** Cluster 2. defined by SNP1 = KCNN3_rs884664 = 2.

SNP2	SNP3	GT^a^	Patients^b^
ANK3_rs2018783	KCNQ3_rs7002144	0 0	22 24 153 212 359 375 553 573 584 585
ANK3_rs2018783	KCNQ3_rs10217015	0 0	22 24 153 212 375 553 573 584 585
ANK3_rs2018783	CNTNAP2_rs1587048	0 0	22 24 305 351 359 421 500 553 573
ANK3_rs2018783	CNTNAP2_rs1524339	0 1	22 153 212 351 359 421 573 584 585
ANK3_rs2018783	CNTN1_rs11178111	0 0	22 24 153 305 351 359 573 584 585
CAMKK2_rs2686343	NCAM1_rs584427	0 0	22 50 154 188 201 351 359 500 524
CAMKK2_rs2686343	SCN2A_rs17184707	0 1	22 91 154 188 201 351 383 417 422
CAMKK2_rs2686343	NFASC_rs10900430	0 1	22 50 91 188 201 212 280 359 422
CAMKK2_rs2686343	NFASC_rs11240304	0 1	22 91 154 188 201 351 383 417 422
CAMKK2_rs2686343	NFASC_rs7535216	0 1	22 91 154 188 201 351 383 417 422
CNTNAP2_rs10808044	BACE1_rs522843	0 0	5 24 78 91 156 178 293 375 421 422 503 553
CNTNAP2_rs10808044	BACE1_rs473210	0 0	5 24 78 91 156 178 293 375 421 422 553
CNTNAP2_rs10808044	BACE1_rs525493	0 0	5 24 78 91 156 178 293 421 422 553
CNTNAP2_rs10808044	BACE1_rs477036	0 0	5 24 78 91 156 178 293 421 422 553
BDNF_rs908867	AQP4_rs3875089	1 0	5 24 50 123 156 176 201 359 378 500
BDNF_rs908867	NRG1_rs2975500	1 0	5 24 50 123 156 176 201 378 500
BDNF_rs908867	KCNQ3_rs17575754	1 0	5 24 50 123 156 176 359 378 500
IMPA2_rs636173	KCNC1_rs7110441	2 0	38 62 74 154 201 422 500 524 526 553
IMPA2_rs636173	KCNC1_rs16934680	2 0	38 62 74 154 201 422 500 524 526 553
CNTNAP2_rs9640245	TNR_rs859437	1 2	27 74 176 189 283 383 500 526 585
CNTNAP2_rs9640245	TNR_rs12119177	1 0	27 74 176 189 283 383 500 526 585
ANK3_rs16914644	KCNN3_rs11264248	1 2	38 62 74 123 147 178 201 305 422
ANK3_rs16914644	KCNN3_rs6695232	1 0	38 62 74 123 147 178 201 305 422
CACNG2_rs2284010	KCNQ3_rs713148	0 2	62 78 86 147 156 176 189 212 359 422 503 526
CACNG2_rs2284010	KCNQ3_rs17595945	0 2	62 78 86 156 176 189 212 359 422 526
KCNC3_rs1559133	PDE4B_rs4288570	1 0	22 62 147 212 280 375 422 500 538
KCNC3_rs1559133	ANK3_rs10821695	1 0	62 147 212 280 293 375 422 526 538
KCNC2_rs2926150	CNTN2_rs11240351	1 1	22 38 78 86 123 176 189 378 503
KCNC2_rs2926150	BACE1_rs522843	1 0	5 47 78 123 156 176 378 417 503
NRCAM_rs11767318	BACE1_rs525493	1 0	5 62 78 178 293 305 421 422 585
NRCAM_rs11767318	BACE1_rs477036	1 0	5 62 78 178 293 305 421 422 585
CAMKK2_rs11065502	SCN2A_rs17184707	1 1	27 91 176 188 201 212 305 359 375 422
CAMKK2_rs11065502	KCNN3_rs11264254	1 1	27 30 156 176 201 305 417 422 524
KCNQ3_rs713148	KCNC2_rs1379963	2 1	47 62 78 86 156 189 421 422 503 585
CNTN1_rs11178111	NRCAM_rs10953566	1 0	27 47 153 176 178 212 359 524 584 585
MBP_rs12962017	PPP2R2C_rs4689408	1 1	5 30 62 135 154 351 378 500 538
KCNN3_rs11264254	P2RX7_rs1718134	2 1	5 38 74 91 188 212 314 421 584
KCNA1_rs1048500	P2RX7_rs503720	0 0	22 47 50 62 135 293 359 417 526
KCNA2_rs3887820	KCNQ2_rs6062929	1 1	24 30 147 153 154 156 188 305 503
IMPA2_rs3859296	PPP2R2C_rs16838658	1 1	22 38 47 176 283 417 524 526 584
NFASC_rs11240304	KCNQ2_rs6090403	1 1	22 91 188 189 229 293 383 417 422
BDNF_rs11030102	SLC12A6_rs17236791	1 1	30 47 74 188 280 293 375 422 538 573
NFASC_rs10900430	KCNQ2_rs6090403	1 1	22 91 188 189 229 293 383 417 422
SCN1B_rs8100085	YWHAH_rs929036	0 2	22 24 74 86 153 154 383 417 538
SPTBN4_rs4803342	MAP2_rs17745941	1 1	30 50 62 78 283 422 500 524 553

Cluster 2 contains 48 patients, 45 combinations of 3 SNP genotypes, and 60 SNP genotypes.

a) GT = genotype for SNP2 and SNP3 (0: Normal homozygote.1: Heterozygote. 2: Variant homozygote); b) Dummy ID.

**Table 3:** Cluster 3.defined by SNP1 = CACNG2_rs2179871 = 2.

SNP2	SNP3	GT^a^	Patients^b^
KCNN3_rs6426998	NCAM1_rs7130671	2 1	44 65 119 146 224 227 232 290 380 417 599 602
KCNN3_rs6426998	KCNC2_rs1458606	2 1	44 65 119 224 290 331 380 417 515 599 602
KCNN3_rs6426998	CAMKK2_rs1653594	2 1	44 119 146 224 227 290 331 380 417 599 602
KCNN3_rs6426998	CAMKK2_rs1140886	2 1	44 65 119 290 331 380 417 515 599 602
KCNN3_rs6426998	CAMKK2_rs1063843	2 1	44 65 119 290 331 380 417 515 599 602
KCNN3_rs6426998	SPTBN4_rs17656504	2 0	44 65 146 224 227 232 290 380 515 602
KCNN3_rs6426998	CNTNAP2_rs6962824	2 1	44 65 119 224 232 290 331 515 602
KCNN3_rs6426998	CNTNAP2_rs2972112	2 1	44 65 146 224 227 380 515 599 602
KCNN3_rs6426998	CNTNAP2_rs7803315	2 0	44 65 119 146 224 227 232 417 602
KCNN3_rs6426998	NFASC_rs6593917	2 1	44 65 146 224 232 290 417 515 599
KCNN3_rs6426998	SCN2A_rs17185905	2 0	44 65 119 146 224 227 232 331 515
KCNN3_rs6426998	ANK3_rs1380452	2 1	44 65 146 224 227 290 417 515 599
KCNN3_rs6426998	CREB1_rs10932201	2 1	44 65 224 232 290 331 417 515 602
CNTNAP2_rs4493828	IMPA2_rs3889500	2 0	65 380 388 417 449 469 484 570 574 599
CNTNAP2_rs4493828	SLC12A6_rs8028501	2 0	65 380 388 417 449 469 484 570 574 599
CNTNAP2_rs4493828	TNC_rs1330351	2 1	65 380 388 417 449 469 484 570 574 599
CNTNAP2_rs4493828	TNC_rs2071520	2 1	65 380 388 417 449 469 484 570 574 599
CNTNAP2_rs4493828	ANK3_rs16914571	2 0	380 388 417 449 469 484 570 574 599
CNTNAP2_rs4493828	AQP4_rs151245	2 1	65 380 388 449 469 484 570 574 599
CNTNAP2_rs4493828	MBP_rs2282557	2 0	65 380 388 417 449 469 484 570 574 599
CNTNAP2_rs4493828	MBP_rs470330	2 0	65 380 388 417 449 484 570 574 599
CNTNAP2_rs4493828	MBP_rs470131	2 0	65 380 388 417 449 484 570 574 599
CNTNAP2_rs4493828	DLG4_rs2586539	2 0	65 380 388 417 449 469 570 574 599
CNTNAP2_rs4493828	KCNC2_rs1880840	2 0	65 380 388 417 449 469 484 570 574
CNTNAP2_rs4493828	SLC12A6_rs16958875	2 0	65 380 388 417 449 484 570 574 599
CNTNAP2_rs2972112	SLC12A6_rs4577050	0 0	44 65 146 224 292 331 449 498 515 522 570
CNTNAP2_rs2972112	SLC12A6_rs436552	0 0	44 146 224 292 331 449 498 515 522
CNTNAP2_rs2972112	PDE4B_rs599381	0 1	44 83 146 227 232 292 420 498 522
CNTNAP2_rs2972112	KCNC1_rs757511	0 2	44 65 119 146 331 415 420 449 498
CREB1_rs2551645	P2RX7_rs6489794	1 1	44 145 227 290 292 330 420 449 484 533
CREB1_rs2551645	ANK3_rs9888033	1 0	44 145 227 265 307 330 380 415 436
PPP2R2C_rs6814782	NRG1_rs4236709	0 0	113 335 415 417 420 436 449 515 533
PPP2R2C_rs6814782	CAMKK2_rs2686343	0 1	44 113 119 290 380 415 515 522 533
CNTNAP2_rs1024676	SPTBN4_rs4803342	0 0	224 265 307 388 415 417 436 533 570 574
CNTNAP2_rs1024676	NRG1_rs2466051	0 1	44 224 330 388 469 504 522 533 599
CACNG2_rs738974	BACE1_rs525493	1 2	65 290 292 335 388 420 504 533 560
CACNG2_rs738974	BACE1_rs477036	1 2	65 290 292 335 388 420 504 533 560
KCNC3_rs636567	PPP2R2C_rs10937735	1 1	44 83 119 146 265 290 335 388 522 533 560
KCNC3_rs636567	CNTNAP2_rs2373289	1 1	83 119 146 335 388 516 522 533 560
CNTNAP2_rs1730399	KCNQ2_rs6090403	1 2	65 113 388 420 449 471 515 516 602
PPP2R2C_rs10937735	SCN2A_rs3769949	1 0	44 113 146 227 265 307 417 471 515 522 533
BACE1_rs525493	SCN2A_rs2060199	2 1	65 290 323 335 388 420 504 533 560
CNTN1_rs1596509	KCNC2_rs1458613	0 1	65 83 145 227 232 330 388 504 533 574
MAP2_rs2663652	KCNN3_rs906280	1 1	224 232 307 331 388 415 482 484 504 515 602
NCAM1_rs2196456	CACNG2_rs926543	0 1	65 145 290 292 330 498 515 516 574
SCN5A_rs7430407	MAP2_rs6733319	1 0	44 146 224 265 335 388 417 436 482
PPP2R2C_rs4386675	KCNN3_rs883319	1 1	83 292 331 388 482 484 497 516 533 574
NFASC_rs16854930	SCN4B_rs678262	1 0	119 265 330 335 388 436 516 533 570
KCNN3_rs7547552	NCAM1_rs1807939	1 0	65 83 145 265 331 388 484 497 515 516 560 574 599

Cluster 3 contains 41 patients, 49 combinations of 3 SNP genotypes, and 65 SNP genotypes.

a) GT = genotype for SNP2 and SNP3 (0: Normal homozygote.1: Heterozygote. 2: Variant homozygote); b) Dummy ID.

**Table 4:** Cluster 4. defined by SNP1 = KCNQ3_rs2469515 = 2.

SNP2	SNP3	GT^a^	Patients^b^
ANK3_rs12049756	SCN2A_rs3769949	1 1	111 268 294 358 360 385 399 444 491 521 538
ANK3_rs12049756	SCN2A_rs997508	1 1	111 294 354 358 360 385 399 444 491 521 538
ANK3_rs12049756	SCN2A_rs12469667	1 1	13 111 294 354 358 360 399 444 491 538
ANK3_rs12049756	ANK3_rs10821702	1 1	13 111 196 268 294 354 360 385 399
ANK3_rs12049756	AQP4_rs9951307	1 0	98 111 196 294 360 385 399 521 538
SCN5A_rs7430407	ANK3_rs1010556	1 1	10 13 20 56 72 196 268 328 336 567
SCN5A_rs7430407	CNTNAP2_rs4431524	1 1	10 20 56 59 72 268 328 336 567
SCN5A_rs7430407	KCNN3_rs11264250	1 1	13 20 56 59 72 268 328 336 567
SPTBN4_rs8107961	TBR1_rs7564766	2 0	0 10 13 18 196 328 343 358 492 527 599
SPTBN4_rs8107961	CAMKK2_rs1140886	2 1	0 18 196 343 358 360 492 527 538 599
ANK3_rs10821677	ANK3_rs12767186	1 1	56 62 98 111 268 294 360 385 399 431 596
ANK3_rs10821677	KCNC1_rs757511	1 2	62 98 111 294 399 444 521 527 596
OLIG2_rs762178	ANK3_rs2393602	2 1	0 20 72 323 328 491 527 538 567 596
OLIG2_rs762178	KCNC2_rs11180386	2 0	0 20 72 323 444 491 492 527 538
CNTNAP2_rs2462603	ANK3_rs10994200	2 0	20 62 72 189 268 354 358 538 567 596
CNTNAP2_rs2462603	ANK3_rs10761454	2 0	20 62 72 189 268 358 538 567 596
PPP2R2C_rs2269920	TRPM2_rs9974831	1 1	0 20 72 111 323 336 343 354 360 521 562
PPP2R2C_rs2269920	KCNC3_rs683856	1 1	196 268 294 354 360 369 492 527 562
MAG_rs1034597	CNTNAP2_rs2972112	1 0	10 13 18 111 294 358 360 385 444 483 538
KCNQ2_rs6089908	NFASC_rs6677763	0 1	294 323 336 343 354 358 492 527 567 596
CNTN1_rs3794247	NRG1_rs4535704	0 1	0 10 62 98 111 294 385 399 491 492
ANK3_rs1380452	TNC_rs1330351	0 1	0 294 328 343 399 444 521 527 567
NRG1_rs2439311	IMPA2_rs662383	1 1	56 111 328 354 360 431 483 567 599
P2RX7_rs7958311	BACE1_rs522843	1 1	59 98 111 189 196 294 360 399 431
CNTNAP2_rs4431524	MBP_rs470826	1 1	20 56 59 72 328 354 358 399 527 596
DLG4_rs507506	SPTBN4_rs814501	0 2	13 18 196 328 360 492 521 538 599
IMPA2_rs628419	KCNQ2_rs6062925	1 2	20 72 98 268 323 336 358 431 527
PPP2R2C_rs3796403	IMPA2_rs3786305	1 1	0 59 98 111 196 336 358 431 483
CNTNAP1_rs2271029	NRCAM_rs6958498	1 1	10 13 196 354 399 492 527 538 562
KCNQ2_rs884851	CNTNAP1_rs9897724	1 0	0 56 294 328 369 385 431 562 596
NRG1_rs3924999	ANK3_rs10761482	1 1	10 13 59 98 294 360 399 431 521
CREB1_rs2551921	MBP_rs9676113	1 1	18 20 72 369 399 483 527 567 599

Cluster 4 contains 37 patients, 32 combinations of 3 SNP genotypes, and 53 SNP genotypes.

a) GT = genotype for SNP2 and SNP3 (0: Normal homozygote.1: Heterozygote. 2: Variant homozygote); b) Dummy ID.

The 156 patients were subdivided into the 4 clusters and into three groups based on three geographic areas in Scandinavia (Oslo, Aarhus, and Copenhagen). The available clinical data were not the same in the three areas. The number of hypomanic, manic and depressive episodes was available from the Norwegian patients, the number of hospital admissions and the presence of alcohol dependence was available from the patients from Copenhagen (Tables 5, 6, 7).

**Table 5:** Number of hypomanic, manic and depressive episodes in patients from Oslo with bipolar disorder.

Cluster 1		Cluster 2		Cluster 3		Cluster 4	
Hypo-manic and manic episodes	Depres-sive episodes	Hypo-manic and manic episodes	Depres-sive episodes	Hypo-manic and manic episodes	Depres-sive episodes	Hypo-manic and manic episodes	Depres-sive episodes
**11**	**20**	**50**	**40**	**39**	**40**	**22**	**22**
**8**	**10**	4	0	**32**	**32**	12	3
6	0	3	0	**30**	**20**	**5**	**15**
4	3	3	0	**25**	**30**	5	5
3	4	2	25	**22**	**21**	3	20
2	0	2	7	**14**	**10**	3	5
1	1	2	3	**13**	**20**	3	2
1	0	2	1	**10**	**51**	2	10
		2	1	**10**	**20**	2	1
		1	1	**7**	**10**	2	1
		1	1	**6**	**6**	1	1
				3	30	1	0
				3	0	1	0
				2	5		
				1	2		
				1	2		
				1	2		
				1	1		
25% with more serious disease		9% with more serious disease		61% with more serious disease		15% with more serious disease	

Each double cell shows the number of hypomanic and manic episodes and the number of depressive episodes for a single patient. The median for the number of hypomanic and manic episodes is 3, and the median for the number of depressive episodes is 3.5. Patients having numbers of episodes above the median for both hypomanic and manic episodes and depressive episodes (bold types) are compared with patients having at least one type of episodes below or equal to the medians. p = 0.0045 for clusters 1+2+4 versus cluster 3 (Fisher's exact test, two-tailed).

**Table 6:** Number of hospital admissions for patients with bipolar disorder in Copenhagen.

Cluster 1	Cluster 2	Cluster 3	Cluster 4
38	38	14	70
25	32	12	41
13	22	8	40
7	17	4	20
7	12	3	19
6	9		11
5	5		7
5	5		7
3	4		6
3	4		
2	3		
1	3		
1	3		
0	2		
	2		
	2		
	0		
	0		

Each box represents one patient.

**Table 7:** Alcohol dependence (1) or non-dependence (0) in patients from Copenhagen with bipolar disorder.

Cluster 1	Cluster 2	Cluster 3	Cluster 4
0	0	0	0
0	0	1	0
0	0	1	0
0	0	1	1
0	0		1
0	0		1
0	0		1
0	0		
0	0		
0	0		
1	0		
1	1		
1	1		
	1		
	1		

Each box represents one patient.

Table 5 shows number of hypomanic and manic episodes and depressive episodes for the single patients. The median for the number of hypomanic and manic episodes is 3, and the median for the number of depressive episodes is 3.5. Patients having numbers of episodes above the median for both hypomanic and manic episodes and depressive episodes (more serious disease) were compared with patients having at least one type of episodes below or equal to the medians (p = 0.0045 for clusters 1+2+4 versus cluster 3).

## Discussion

The four clusters of combinations of SNP-genotypes were statistically significantly associated with bipolar disorder; whereas biological or clinical significance of the clusters was not apparent, apart from the original selection of genes related to signal transduction [3]. These genes are shown in Table 8. The relatively little overlap between the patients in the clusters led to an analysis of available clinical data from the psychiatric departments that had recruited the patients from three locations in Scandinavia. The division of patients according to locations, clusters and availability of clinical data led to the small groups of patients shown in Tables 5–7.

**Table 8:** Selected genes and function [3].

Gene	Location	Name and/or Function
*ANK3*	10q21	Role for structure and function of nodes of Ranvier
*AQP4*	18q11.2–q12.1	Regulator of vasopression secretion
*ATP1A2*	1q21–q23	Na+/K+ ATPase alpha-2 subunit
*ATP1A3*	19q13.31	Na+/K+ ATPase alpha-3 subunit
*AVPR1B*	1q32	Arginine vasopressin receptor 1B
*BACE1*	11q23.2–q23.3	Regulation of the voltage dependent Na-channels.
*BDNF*	11p13	Involved in neuroplasticity and stress response
*CACNG2*	22q13.1	Neuronal calcium channel gamma subunit, stabilize the channel in an inactive state
*CAMKK2*	12q24.2	Involved in activation of CREB1
*CLDN11*	3q26.2–q26.3	Role in myelinisation
*CNTN1*	12q11–q12	Cell adhesion molecule
*CNTN2*	1q32.1	Cell adhesion molecule
*CNTNAP1*	17q21	Contactin-associated protein, may be the signaling subunit of contactin
*CNTNAP2*	7q35–q36	Cluster voltage-gated potassium channels, localized at the juxtaparanodes
*CREB1*	2q34	Transcription factor
*DLG4*	17p13.1	Neuronal development, recruited into potassium channel clusters
*ERBB4*	2q33.3–q34	Neuregulin-1 receptor, involved in mitogenesis and differentiation
*GSK3B*	3q13.3	Neuronal cell development (Related to lithium respons)
*IMPA2*	18p11.2	Inositol monophosphatase (Related to lithium respons)
*KCNA1*	12p13.32	Voltage-gated delayed potassium channel
*KCNA2*	1p13	Voltage-gated delayed potassium channel, delayed rectifier class
*KCNC1*	11p15	Mediates the voltage-dependent potassium ion permeability of excitable membranes
*KCNC2*	12q14.1	Mediates the voltage-dependent potassium ion permeability of excitable membranes
*KCNC3*	19q13.3–q13.4	Mediates the voltage-dependent potassium ion permeability of excitable membranes
*KCNN3*	1q21.3	Potassium conductance Ca-activited channel, regulate neuronal excitability
*KCNQ2*	20q13.3	Voltage-gated potassium channel plays a role in the regulation of neuronal excitability
*KCNQ3*	8q24	Voltage-gated potassium channel plays a role in the regulation of neuronal excitability
*MAG*	19q13.1	Central role i myelinisation, involved in myelin-neuron cell-cell interactions
*MAP2*	2q34–q35	Microtubule-associated protein, involved in neurogenesis
*MBP*	18q23	Major constituent of the myelin sheath of oligodendrocytes in the nervous system
*MCHR1*	22q13.2	Inhibit cAMP accumulation stimulate intracellular Ca-flux
*MCTP2*	15q26.2	Intercellular signal transduction
*MOG*	6p22.1	Involved in completion and maintenance of the myelin sheath and in cell-cell communication
*NCAM1*	11q23.1	Neural cell adhesion molecule 1
*NFASC*	1q32.1	Cell adhesion; organization of the axon initial segment (AIS) and nodes of Ranvier
*NRCAM*	7q31.1–q31.2	Ankyrin-binding protein is involved in neuron-neuron adhesion
*NRG1*	8p12	Associated with ERBB receptors
*NTRK1*	1q21–q22	Neurotrophic tyrosine kinase, receptor, type 1
*OLIG2*	21q22.11	Oligodendrocyte lineage transcription factor 2
*P2RX7*	12q24	Ligand-gated ion channel
*PDE4B*	1p31	Phosphodiesterase 4B, cAMP-specific
*PPP2R2C*	4p16.1	Protein phosphatase 2, regulatory subunit B, gamma isoform
*SCN1B*	19q13.1	Sodium channel beta subunit, propagation of nerveimpulses, binding to contactin
*SCN2A*	2q23–q24	Sodium channel alpha subunit, generation and propagation of action potentials in neurons
*SCN2B*	11q23	Sodium channel, voltage-gated, type II, beta
*SCN4B*	11q23.3	Sodium channel, voltage-gated, type IV, beta
*SCN5A*	3p21	Sodium channel, voltage-gated, type V, alpha subunit
*SCN8A*	12q13	Sodium channel, voltage gated, type VIII, alpha subunit, associated with ANK3
*SLC12A6*	15q13–q15	Electroneutral potassium-chloride cotransporter 3
*SPTBN4*	19q13.13	Involved in location of specific membrane proteins in polarized regions of neurons
*TBR1*	2q24	Transcription factor, critical for early cortical development
*TNC*	9q33	Regulation of Na channels. Interaction with CNTN1
*TNR*	1q24	Extracellular matix protein expressed primarily in the central nervous system
*TRPM2*	21q22.3	Transient receptor potential cation channel, subfamily M, member 2
*YWHAH*	22q12.3	Tyrosine 3-monooxygenase/tryptophan 5-monooxygenase activation protein, eta polypeptide

Using numbers of hypomanic, manic and depressive episodes higher than the median for these episodes as an indication of severity of disease, it was found that the number of patients with more severe disease was higher in one cluster compared with the three other clusters (Table 5). This result suggests that it may be possible to connect combinations of genetic data to clinical data. The figures in Table 6 and 7 may led to similar suggestions, but although significant differences may be found between the distributions in these tables, the statistical power is low and no significant results may remain after correction for multiple testing.

Due to the relatively low number of patients as well as of clinical data, no strong conclusions can be drawn from this study. However, the results in Table 5 indicated that some genetic subgroups may be more affected by their illness than other subgroups, hereby justifying further work with combinations of genetic data as a method to connect genetic and clinical data. Hopefully, other studies with more patients, more genetic data and more clinical data will try to look at combinations of their data.

## Materials and Methods

The patient sample, genes, SNP selection and genotyping, statistics and data processing regarding Table 1, 2, 3, 4 were described previously [3]. The Norwegian Scientific-Ethical Committees, the Norwegian Data Protection Agency, the Danish Scientific Committees, and the Danish Data Protection Agency approved the study. All patients gave written informed consent prior to inclusion in the project. The data in Table 5 were analyzed statistically with Fisher's exact test, two-tailed. In Tables 5, 6, 7 each box represents one patient. The numbers of hypomanic, manic, and depressive episodes in Norwegian patients were obtained by SCID [8]. The numbers of admissions in Copenhagen were obtained from patient records. Patients from Copenhagen were diagnosed with alcohol dependence when the patient was, or had been, treated in an alcohol clinic.
