# Correction: Connection between Genetic and Clinical Data in Bipolar Disorder

**DOI:** 10.1371/annotation/20489174-1069-436f-86e8-bc964abee1ba

**Published:** 2013-05-08

**Authors:** Erling Mellerup, Ole Andreassen, Bente Bennike, Henrik Dam, Srdjan Djurovic, Thomas Hansen, Ingrid Melle, Gert Lykke Møller, Ole Mors, Pernille Koefoed

There was an error in the fifth author's name.

The correct name is: Srdjan Djurovic

The correct citation is: Mellerup E, Andreassen O, Bennike B, Dam H, Djurovic S, et al. (2012) Connection between Genetic and Clinical Data in Bipolar Disorder. PLoS ONE 7(9): e44623. doi:10.1371/journal.pone.0044623 

